# Microbiome dysbiosis is associated with disease duration and increased inflammatory gene expression in systemic sclerosis skin

**DOI:** 10.1186/s13075-019-1816-z

**Published:** 2019-02-06

**Authors:** Michael E. Johnson, Jennifer M. Franks, Guoshuai Cai, Bhaven K. Mehta, Tammara A. Wood, Kimberly Archambault, Patricia A. Pioli, Robert W. Simms, Nicole Orzechowski, Sarah Arron, Michael L. Whitfield

**Affiliations:** 1 Department of Molecular and Systems Biology, Geisel School of Medicine at Dartmouth, Hanover, NH USA; 2 Program in Quantitative Biomedical Sciences, Geisel School of Medicine at Dartmouth, Hanover, NH USA; 3 Department of Microbiology and Immunology, Geisel School of Medicine at Dartmouth, Hanover, NH USA; 4 Department of Biomedical Data Science, Program in Quantitative Biomedical Sciences, Geisel School of Medicine at Dartmouth, Hanover, NH USA; 5 Division of Rheumatology, Dartmouth-Hitchcock Medical Center, Lebanon, NH USA; 6 Division of Dermatology, University of California, San Francisco, USA; 7 Division of Rheumatology, Arthritis Center, Boston University Medical Center, Boston, MA USA; 8 Department of Environmental Health Science, University of South Carolina Arnold School of Public Health, Columbia, SC USA

**Keywords:** Microbiome, Systemic sclerosis, Scleroderma, Metagenomics, RNA-sequencing

## Abstract

**Background:**

Infectious agents have long been postulated to be disease triggers for systemic sclerosis (SSc), but a definitive link has not been found. Metagenomic analyses of high-throughput data allows for the unbiased identification of potential microbiome pathogens in skin biopsies of SSc patients and allows insight into the relationship with host gene expression.

**Methods:**

We examined skin biopsies from a diverse cohort of 23 SSc patients (including lesional forearm and non-lesional back samples) by RNA-seq. Metagenomic filtering and annotation was performed using the Integrated Metagenomic Sequencing Analysis (IMSA). Associations between microbiome composition and gene expression were analyzed using single-sample gene set enrichment analysis (ssGSEA).

**Results:**

We find the skin of SSc patients exhibits substantial changes in microbial composition relative to controls, characterized by sharp decreases in lipophilic taxa, such as *Propionibacterium*, combined with increases in a wide range of gram-negative taxa, including *Burkholderia*, *Citrobacter*, and *Vibrio*.

**Conclusions:**

Microbiome dysbiosis is associated with disease duration and increased inflammatory gene expression. These data provide a comprehensive portrait of the SSc skin microbiome and its association with local gene expression, which mirrors the molecular changes in lesional skin.

**Electronic supplementary material:**

The online version of this article (10.1186/s13075-019-1816-z) contains supplementary material, which is available to authorized users.

## Introduction

Systemic sclerosis (SSc) is a progressive autoimmune disease that results in inflammation, fibrosis, and dysfunction of multiple organ systems including the skin, lungs, gastrointestinal tract, and blood vessels. Recent advances have provided significant insight into the molecular and immunologic changes characteristic of SSc patients [1–5]; however, the underlying mechanisms that initiate and perpetuate disease pathologies remain poorly understood.

Evidence for dysbiosis as a source of disease pathology is well-documented in inflammatory skin conditions, such as psoriasis, where patients exhibit significant increases in both *Propionibacterium* and *Staphylococcus* on lesional skin [6]. In atopic dermatitis (AD), patients exhibit temporal shifts in skin microbiome composition, with microbiome diversity decreasing during disease flares, characterized by significant increases in *Staphylococcus* levels, followed by increased diversity thereafter [7]. These patterns of dysbiosis suggest a mechanism by which relative changes in the abundance of specific taxa directly influence disease pathology [7].

A wide array of potential etiologic agents have been proposed for SSc, including viruses, bacteria, and fungi. Viruses such as cytomegalovirus (CMV), parvovirus B19, Epstein-Barr virus (EBV), and endogenous retroviruses have all been postulated as potential triggers of SSc [8–10]. EBV transcripts have been reported in lesional skin of SSc patients [11]. Among bacteria, *Helicobacter pylori* has been implicated in the etiology and progression of numerous autoimmune diseases, though its role in SSc remains controversial with studies both confirming and refuting such an association [10, 12, 13]. The most recent addition to the list of potential etiologic agents is the fungus *Rhodotorula glutinis*, a ubiquitous environmental contaminant and occasional skin commensal, which was found to be strongly associated with lesional skin of early-stage, untreated diffuse SSc patients [14].

Here, we examined skin biopsies from a diverse cohort of SSc patients and healthy controls by RNA-sequencing (RNA-seq) to obtain an unbiased assessment of the SSc microbiome and its relationship with patient gene expression. We show a reproducible shift in microbiome composition characterized by decreases in lipophilic taxa, along with increases in a variety of gram-negative bacteria that mirror local changes in inflammatory gene expression. These changes are closely tied to underlying gene expression associated with lipid signaling and immune activation. Genus-level taxonomic changes were associated with disease duration and the inflammatory intrinsic gene expression subset. Together, these data demonstrate that the skin microbiome composition in SSc mirrors molecular pathogenesis.

## Methods

### Patient selection

Study participants provided written, informed consent prior to sample collection in accordance with the Declaration of Helsinki Protocol and the Institutional Review Boards of Boston University Medical Center, Boston, MA, Dartmouth-Hitchcock Medical Center, Lebanon, NH, and the Hospital for Special Surgery, New York, NY. All patients met the American College of Rheumatology classification criteria for SSc [15], with further classification as either diffuse [16] (dSSc) or limited [17] (lSSc) disease. SSc patients with disease duration < 2.5 years were classified as “early stage” and patients with disease duration > 8 years were classified as “late stage” for this analysis.

### Biopsy processing and RNA-seq

Lesional forearm and, for a subset of patients, non-lesional back skin was collected by punch biopsy (4 mm) from 15 SSc patients and 6 healthy volunteers. An additional 8 baseline samples collected as part of a Nilotinib clinical trial [18] were also included in this analysis. Following collection, samples were immediately placed in RNA*Later* (Life Technologies, Carlsbad, CA) at 4 °C overnight, followed by − 80 °C until needed. Tissue homogenization was performed using the Qiagen TissueLyser II (Qiagen, Gaithersburg, MD). RNA extraction was performed using the Qiagen RNeasy Fibrous Tissue Mini Kit run on the QIAcube (Qiagen). RNA concentration and RNA integrity were assessed using the Agilent 4200 TapeStation (Agilent, Santa Clara, CA). RNA-seq libraries were generated from 100 ng total RNA prepared using the Illumina TruSeq Stranded Total RNA Library Prep Kit with Ribo-Gold rRNA depletion (Illumina, San Diego, CA). Samples were then multiplexed and sequenced on an Illumina NextSeq 500 sequencer, producing an average of 80–100 million 75-bp paired-end reads per sample.

### Human gene expression analyses

Raw sequencing reads were aligned to the human genome (hg19) using STAR aligner [19] and expressed as fragments per million mapped reads (FPKM). Designation of intrinsic molecular subsets for SSc patients was performed using a gene-specific normalization method to render RNA-seq values distributions similar to microarray so that supervised machine learning algorithms can be applied regardless of the platform used to generate data, as described [20]. Normalized RNA-seq data were classified using a support vector machine trained using a merged and curated dataset composed of samples from GSE9285, GSE32413, and GSE45485. To visualize results, the probe ID list from Johnson et al. [4] was collapsed on gene ID. This gene list was compared against normalized FPKM values for all 36 RNA-seq samples, resulting in a total of 1010 overlapping genes; a full list of all genes and normalized expression values is shown in Additional file 1: Table S1. Data were then hierarchically clustered using Cluster 3.0 [21] and visualized using Java TreeView [22].

### Metagenomic filtering and microbiome annotation

Metagenomic filtering and microbiome annotation was run using the Integrated Metagenomic Sequencing Analysis (IMSA) software package [23] and compared against the National Center for Biotechnology Information (NCBI) non-redundant nucleotide database (minimum significance = 1 × 10^− 15^), followed by a secondary BLAST alignment against the NCBI viral genome repository (minimum significance = 1 × 10^− 5^). To limit inclusion of spurious hits, sample annotation was limited to sequences mapping to five or fewer species, with ties split equally across species. Outputs were then filtered based on taxonomy to include only archaea, bacteria, fungi, and viruses. Normalization of taxonomic outputs was performed by rounding down to the nearest integer and rarefying to the level of the depth of the lowest sample using the Quantitative Insights Into Molecular Ecology (QIIME) platform [24]. Batch effects associated with library preparation were removed by median centering across taxa. Statistical analyses were performed using Statistical Package for the Social Sciences (SPSS) software (IBM, version 23); additional analyses, including corrections for multiple hypothesis testing using the method of Benjamini & Hochberg [25], were performed in R.

### Pathway activation and microbiome abundance

Single-sample gene set enrichment analysis (ssGSEA) [26] was run as a module in GenePattern, using relevant KEGG pathways as the query gene sets. A correlation matrix was then generated by calculating Pearson’s correlations for all combinations of ssGSEA values and genus-level abundance across all patients. Data were then hierarchically clustered using Cluster 3.0 and visualized using Java TreeView.

## Results

### Patient characteristics

Lesional forearm skin biopsies were collected from 23 SSc patients; seven patients also provided biopsies of non-lesional back skin. Forearm skin biopsies were also obtained from 6 age- and gender-matched healthy controls. Samples were collected from three independent clinical centers and included both clinically limited (lSSc) and diffuse (dSSc) disease, with disease duration ranging from 0 to 35 years. The patient population consisted primarily of early-stage patients (disease duration ≤ 2 years), though a handful of very late-stage patients (disease duration > 10 years) were also included to assess microbiome changes over time. Clinical information on these patients is summarized in Table 1; a full breakdown of patient clinical information is presented in Additional file 2: Table S2. Assessments of skin involvement were determined based on overall modified Rodnan skin score (mRSS), as local scores were not available for all patients. No significant differences in age, sex, or race were evident between SSc and controls (*p* > 0.05 for all).

**Table 1:** Summary clinical information

	Control subjects	SSc patients
	(*N* = 6)	(*N* = 23)
Age, median (range) years	53 (25–67)	53 (27–77)
Sex, *N* (%) female	4 (67%)	19 (83%)
Race, *N* (%) Caucasian	5 (83%)	20 (87%)
SSc subtype, *N* (%) diffuse	NA	15 (65%)
MRSS, median (range)	NA	16 (0–44)
Disease duration from first non-Raynaud’s, median (range) years	NA	1.0 (0–35)
ILD/PAH, *N* (%)	NA	8 (35%)
ANA primary pattern, *N* (%) patients		
Homogenous	NA	1 (4%)
Nucleolar	NA	5 (22%)
Speckled	NA	6 (26%)
Centromere	NA	2 (9%)
SSc-specific antibodies, *N* (%)		
Anti-centromere	NA	3 (13%)
Scl-70	NA	3 (13%)
RNA polymerase III	NA	5 (22%)
Current therapies, *N* (%)	NA	17 (74%)
Prior therapies, *N* (%)		
Amlodipine	NA	4 (17%)
Methotrexate	NA	4 (17%)
Prednisone	NA	3 (13%)

Abbreviations: SSc, systemic sclerosis; ANA, anti-nuclear antibodies; MRSS, modified Rodnan skin score; ILD, interstitial lung disease; PAH, pulmonary arterial hypertension; NA, not applicable

Current and prior therapies include all treatments observed in three or more patients

### Sequencing and annotation

**Fig. 1:**
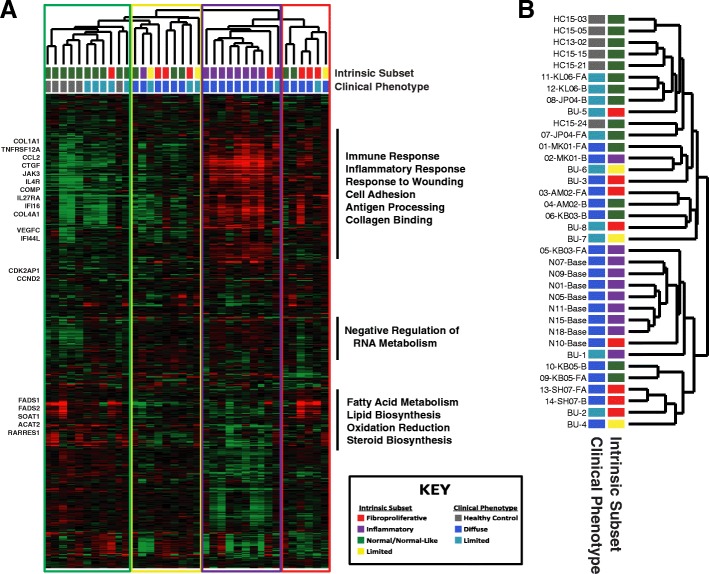
Intrinsic subset analysis of RNA-seq reads from SSc skin. **a** Assignment of intrinsic molecular subsets for SSc patients was performed using a support vector machine (SVM) developed for the purpose (Franks et al. In Press). Displayed are the 1010 genes from Johnson et al. [4] collapsed on gene ID and extracted from the normalized FPKM values for all 36 RNA-seq samples. Hierarchical clustering revealed distinct molecular subsets of disease, consistent with previous publications [1–3]. The sample dendrogram is colored to indicate intrinsic subset designations: normal-like (green), limited (yellow), inflammatory (purple), proliferative (red). **b** Hash marks indicate SSc clinical diagnosis associated with each sample. Black bars indicate genes that clustered together hierarchically; the most significantly overrepresented GO terms are listed

RNA-seq was performed on 36 skin biopsies, from 29 unique patients, resulting in an average of 83 million reads per sample (range 51,278,817–112,643,430). Raw sequencing reads were aligned to the human genome (hg19) using STAR aligner [19], and the expression level of each gene was expressed as fragments per million mapped reads (FPKM). Intrinsic gene expression subset designations were determined based on support vector machine classification using normalized FPKM values [20]. Hierarchical clustering using the gene list from Johnson et al. [4], resulting in a total of 1010 overlapping genes, revealed distinct molecular subsets of disease, characterized by strong immune activation, lipid signaling, and proliferation signals, consistent with previous publications [1–3] (Fig. 1a; Additional file 1: Table S1). Together, these data suggest our patient cohort is representative of the four major intrinsic gene expression subsets of SSc. Additionally, we find that forearm and back samples largely tend to cluster together, consistent with previous analyses (Fig. 1b) [27].

Filtering of human sequence reads and microbiome annotation was performed using Integrated Metagenomic Sequence Analysis (IMSA) [23], yielding an average of 18,794 informative hits, defined as sequences mapping to five or fewer species, per skin biopsy (range 3098–74,429) across 1870 genera. To adjust for library-specific effects, all data were rarefied to the level of the lowest sample, followed by median centering of each genus by library preparation batch. This approach substantially reduced batch effects associated with library preparation, enabling direct comparisons of sample outputs across patients.

### Antimicrobial gene expression is suppressed in SSc lesional skin

Antimicrobial peptides (AMPs), including cathelicidin (CAMP/LL-37), α-defensins, and β-defensins, are an essential component of epithelial barrier defenses. To assess the role of AMPs in SSc, we compared gene expression levels between SSc and controls, as well as between lesional forearm and non-lesional back skin. Among the major AMPs, dermcidin (DCD) is highly expressed across samples, regardless of disease type, while other major AMPs, including cathecidin (CAMP) and the α-defensins, were virtually undetected, with no difference in expression between SSc and controls. In contrast, β-defensin 1 (DEFB1), an AMP produced by epithelial cells, is expressed across all samples; however, these levels are significantly lower is SSc lesional skin compared to healthy controls (*p* < 0.001 by unpaired *t*-test), as well as in lesional forearm compared to non-lesional back skin (*p* = 0.007 by paired *t*-test) (Additional file 3: Table S3). Similar results were also seen between SSC lesional skin and healthy controls in a previous SSc skin RNA-seq dataset (Additional file 3: Table S3), suggesting a potential mechanism underlying microbiome differences in SSc patients.

### Microbiome genus-level differences are correlated with SSc clinical phenotypes

**Fig. 2:**
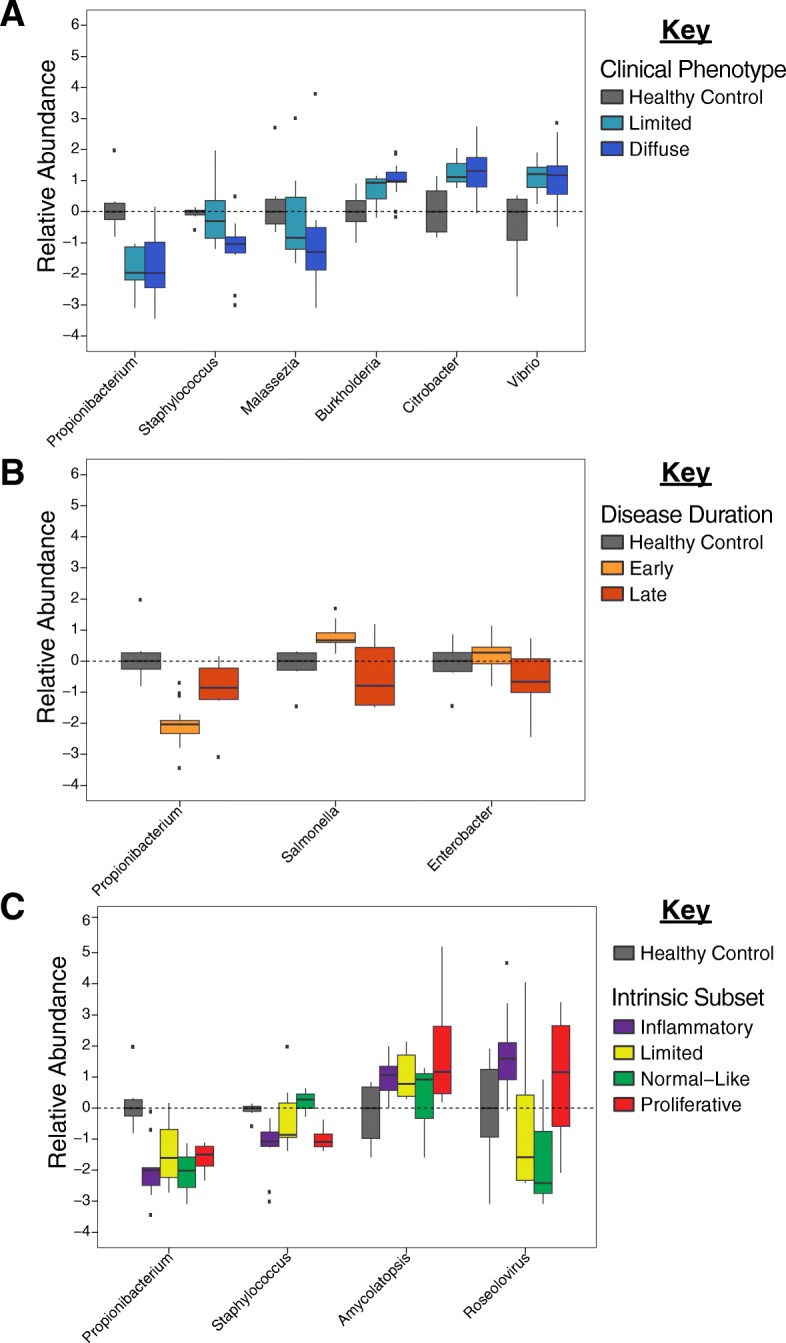
Differential abundance of major skin taxa. SSc lesional skin exhibits significant changes in microbiome composition, relative to controls. Differential abundance of select genera, relative to controls, based on **a** clinical subtype, **b** disease duration (early, < 5 years; late, > 5 years), and **c** intrinsic molecular subset [1]

SSc patients exhibited large changes in microbiome composition relative to controls, characterized by decreases in lipophilic taxa, such as *Propionibacterium* and *Staphylococcus*, combined with increases in a wide range of Gram-negative bacteria, including *Burkholderia*, *Citrobacter*, and *Vibrio* (*p* < 0.05 for all; Fig. 2a; Additional file 4: Table S4). These differences were not associated with clinical subtype, with limited and diffuse disease exhibiting broadly similar abundances of major taxa (Fig. 2a). Decreases were also observed in the fungus *Malassezia* relative to controls, with the greatest decrease occurring in dSSc patients.

Associations between disease duration and genus-level abundance were also evident, with significant (*p* < 0.05) or near-significant (*p* < 0.10) differences in 6 of the top 21 genera, including *Propionibacterium*, *Salmonella*, and *Enterobacter* (Fig. 2b; Additional file 4: Table S4). Relative decreases in *Propionibacterium* were evident for both early- and late-stage patients, relative to controls. We observed differential directions for the relative abundance of *Salmonella* including significant increases for early-stage patients and reduced abundance in late-stage patients. Comparisons between the four intrinsic molecular subsets of disease revealed modest differences associated with the normal-like and inflammatory subsets, with normal-like patients broadly mimicking differences seen between SSc and controls, while the inflammatory group was characterized by decreased *Staphylococcus* and increased *Roseolovirus*, relative to other subsets (Fig. 2c). The absence of more acute genus-level distinctions between subsets is likely the result of high levels of some genera in both inflammatory and proliferative patients (Fig. 2c), thereby limiting the diagnostic value of any single genus. Other clinical cofactors, including sex and autoantibody status, were not statistically different between groups. A full list of comparisons for each clinical cofactor is shown in Additional file 4: Table S4.

### Core microbiome by patient is predictive of clinical involvement

**Fig. 3:**
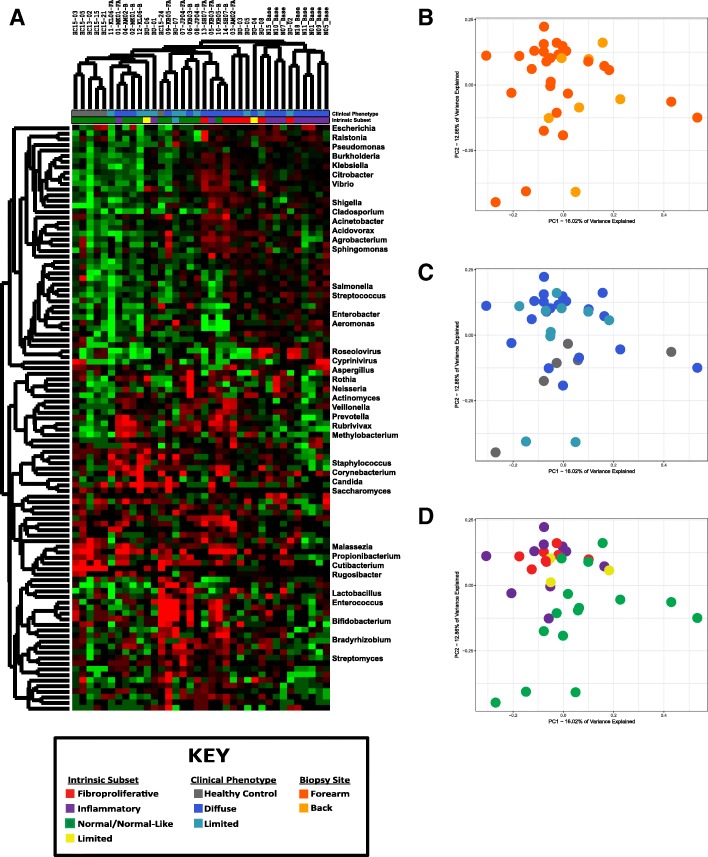
Distribution of the SSc skin core microbiome. The distribution and relative abundance of the SSc skin core microbiome was calculated by rarefaction to the depth of the lowest sample, and filtering to retain the fewest taxa necessary to account for 90% of all reads, resulting in a total of 103 unique genera. Data were then log_2_-transformed and median centered by library preparation. **a** Hierarchical clustering of the core microbiome. Hash marks below the dendrogram indicate intrinsic subset designations and SSc clinical diagnosis for each sample. Principal component analysis of the core microbiome was performed to identify associations between microbiome composition and **b** biopsy location, **c** clinical diagnosis, and **d** intrinsic subset

To identify changes in microbiome composition associated with clinical covariates, we calculated the number of taxa that accounted for 90% of the annotated reads across our entire dataset, which we collectively refer to as the SSc skin core microbiome. The SSc skin core microbiome was composed of 103 genera and included representatives from bacteria, fungi, and viruses. Organisms not included in the core microbiome were exclusively low abundance taxa found in only a small number of samples. Hierarchical clustering of the SSc skin core microbiome revealed patterns of microbial abundance closely mimicking that seen within an individual, characterized by clear differences between SSc and controls (Fig. 3a). Organisms of the SSc skin microbiome formed distinct branches within the dendrogram. Lipophilic commensals (*Malassezia*, *Propionibacterium*, and *Cutibacterium*) were the predominant genera in normal-like patients, Gram-negative bacteria (*Veillonella*, *Prevotella*, *Neisseria*, and *Actinomycetes*) were abundant in the limited and proliferative subsets, and viruses (*Roseolovirus* and *Cyprinivirus*) were highest in inflammatory patients (Fig. 3a). These patterns are consistent with the various environmental niches associated with each class of organisms and are suggestive of changes in skin morphology and immune activation associated with each subset.

SSc skin microbiome profiles were analyzed using principal component analysis (PCA) to identify the broad, population-based changes associated with clinical covariates. Lesional forearm and non-lesional back skin were not significantly different among SSc patients (*p* = 0.097; Fig. 3b; Additional file 5: Figure S1). Similarly, no significant differences were evident based on SSc clinical subtype (*p* = 0.156; Fig. 3c) or mRSS at the time of biopsy (Additional file 6: Figure S2). In contrast, microbiome profiles were strongly correlated with intrinsic subset, with the strongest differences seen in normal-like and inflammatory patients, indicative of a link between disease activity of microbial abundance (*p* = 0.014; Fig. 3a, d).

### Microbiome composition is correlated with inflammatory pathway activation in SSc skin biopsies

**Fig. 4:**
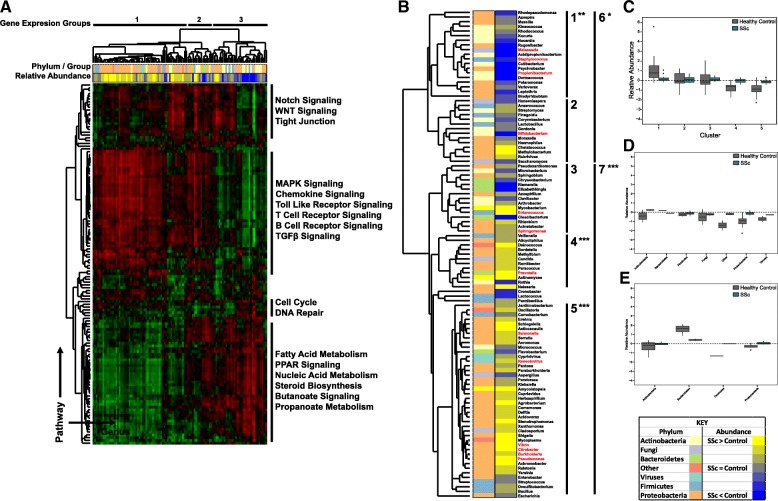
Microbiome composition is associated with pathway activation in SSc skin. Single-sample gene set enrichment analysis (ssGSEA) was run against normalized FPKM values for all 36 patient samples, using curated KEGG pathways as the probe gene sets. A correlation matrix was then generated by calculating Pearson’s correlations for all combinations of ssGSEA values and genus-level abundance across all patients. **a** Hierarchical clustering of the correlation matrix revealed strong associations between SSc-associated gene expression pathways and microbial composition. **b** Taxonomic clustering based on gene expression. Hash marks indicate phylum/group associated with each sample. Relative abundance indicates the degree to which each genus is differentially present in SSc patients, relative to controls with yellow indicating abundance is higher in SSc, while blue indicates abundance is higher in controls. Black bars indicate KEGG pathways that clustered together hierarchically, with representative pathways listed alongside each cluster (*p < 0.05; ** *p* < 0.01; *** *p* < 0.001 by paired *t*-test). Clinically relevant genera are highlighted in red. **c** Relative abundance of all genera by taxonomic cluster. **d**, **e** Distribution of taxa is shown for cluster 5 (**d**) and cluster 3 (**e**)

Given the close association seen between clinical subtype and microbiome composition, we next sought to identify relationships between relevant molecular pathways and taxonomic abundance using single-sample gene set enrichment analysis (ssGSEA). ssGSEA analysis generates a single value quantifying the extent to which a given gene set is coordinately up- or downregulated in a sample. This analysis was repeated for all available KEGG pathways, generating a table of pathway activation scores for each patient sample (Additional file 7: Table S5). Using this data, we then used Pearson’s correlations to compare each of these individual pathways against all genera in the SSc skin core microbiome (Additional file 8: Figure S3). The resulting correlation matrix allows for a direct comparison of gene expression and microbiome composition (Fig. 4a).

Hierarchical clustering of this dataset revealed strong associations between human gene expression and microbial abundance. Processes such as T cell, B cell, chemokine, and transforming growth factor beta (TGFβ) signaling in the absence of fatty acid signaling are strongly indicative of the inflammatory subset (Fig. 4a, cluster 1). Lower immune activation signals in combination with major fatty acid metabolism processes are commonly seen in the proliferative subset (Fig. 4a, cluster 2), while fatty acid signaling in the absence of immune activation is most commonly seen in normal-like patients (Fig. 4a, cluster 3).

Taxonomic abundance was strongly associated with the molecular processes of immune activation, lipid metabolism, cell proliferation, and Notch/Wnt signaling (Fig. 4a). Clustering of these processes was strongly correlated with differences in microbial abundance between SSc and controls, with statistically significant differences evident in 5 of 7 clusters (paired t-test, *p* < 0.05 for all; Fig. 4a, b). Among the most significant clusters was cluster 1, dominated by major lipophilic taxa, such as *Malassezia* and *Propionibacterium*, along with numerous Gram-positive Actinobacteria species (Additional file 9: Figure S4). These organisms were significantly more abundant in healthy controls (*p* < 0.001 for Actinobacteria and *Propionibacterium* by paired *t*-test) and exhibited strong positive correlations to lipid metabolism and cell proliferation KEGG pathways (Fig. 4a, b). In contrast, cluster 5 exhibits substantial increases in a wide range of Proteobacteria and other Gram-negative taxa in SSc patients (*p* < 0.001 by paired *t*-test) and is strongly correlated with KEGG immune activation pathways, including Toll-like receptor (TLR) and TGFβ signaling (Fig. 4a, d). Cluster 3 shows strong, positive correlations with immune activation, lipid metabolism, and Notch/Wnt signaling and is associated with statistically significant decreases in Bacteroidetes levels in SSc patients (*p* = 0.028 by paired *t*-test), combined with modest increases in Proteobacteria, relative to controls (*p* = 0.085 by paired *t*-test; Fig. 4a, e). These data demonstrate a strong association between underlying gene expression and the composition of the skin microbiome in SSc.

## Discussion

Recent studies have provided significant insight into the immunologic and gene expression-based changes characteristic of SSc patients; however, the underlying mechanisms that initiate and perpetuate disease pathologies remain poorly understood. Analyses of the skin [14] and gut [28] of SSc patients have revealed substantial changes in microbiome composition (dysbiosis), though the role of these organisms in disease pathology is not known. Here, we examined skin biopsies from a diverse cohort of SSc patients by RNA-seq, allowing for the unbiased metagenomic analysis of all potential pathogens, including bacteria, fungi, and viruses, as well as providing a platform from which to investigate the relationship between microbiome composition and underlying gene expression. Limitations of this study include the small number of samples and incomplete clinical data for some patients including a lack of local skin scores. The bimodal distribution of disease duration in our cohort is a confounding factor when assessing changes in microbiome composition over time.

Previous studies examining the composition of the bacterial microbiome in healthy individuals identified three basic environments, dry, moist, and sebaceous, which are reflected in the bacterial populations of these regions [29]. Sebaceous regions, such as the face and back, harbored large proportions of *Propionibacterium* and *Staphylococcus* species, while dry regions, such as the forearm, show a shift towards lower levels of *Propionibacterium* in combination with higher percentages of *Proteobacteria*, *Corynebacteria*, and *Flavobacteriales* [29]. When considered in this context, where the bacterial microbiome changes as a function of local lipid and moisture levels, the data presented here paint a picture of SSc as a more extreme version of this process, with disease-specific anatomical changes playing a major role in shaping microbiome composition. Lesional SSc forearm skin exhibited significant decreases in *Propionibacterium* and *Staphylococcus* levels relative to controls, along with increases in a wide range of Proteobacteria (Fig. 2), a continuation of the normal differences seen between sebaceous and dry regions of healthy controls. The underlying basis for these changes is also evident at the molecular level, with abundance of lipophilic taxa, such as *Propionibacterium* and *Malassezia*, strongly associated with fatty acid metabolism and lipid signaling pathways, while Proteobacteria were more elevated in patients with active immune signaling (Fig. 4). Microbiome profiles were not significantly different between lesional forearm and non-lesional back skin in this study, with paired samples clustering strongly based on patients, with minimal effect seen in terms of the anatomical site (Additional file 5: Figure S3). This observation further implicates the underlying gene expression as a major driver of microbiome composition, as disease-related changes in gene expression are consistent between SSc lesional forearm and non-lesional back, yet the microbiome profiles of these sites are strongly divergent in healthy controls.

From a mechanistic standpoint, changes in the SSc skin microbiome may be attributed to physical changes associated with fibrotic skin. Atrophy of both hair follicles and sebaceous glands is commonly seen in SSc patients [30], resulting in the loss of both an essential food source, as well as the physical niche where many of these species reside [31]. This loss of skin appendages leads to a weakening of the acid mantle, and a loss of skin barrier function [32].

The strong association between increased Gram-negative taxa, particularly Proteobacteria, and immune activation shown here suggests a potential link between the skin microbiome and immune activation. Analysis of host-microbiome interactions, particularly with the host immune system, is necessary to determine the extent to which these organisms are capable of exacerbating and perpetuating the inflammatory responses in SSc skin.

In a preliminary study of the skin microbiome, increased levels of *R. glutinis* were detected on lesional skin of four untreated, early-stage patients, with only background levels seen in controls, suggesting a potential link between disease etiology and the skin microbiome [14]. Unfortunately, a direct assessment regarding the etiologic nature of this organism was not possible here due to differences in the two patient cohorts, both in terms of prior treatment and disease duration. The majority of early-stage patients described here were not receiving immunosuppressive therapy at the time of biopsy, though all were receiving treatment for SSc-associated symptoms, including vascular symptoms and gastrointestinal reflux. In contrast, five of six late-stage patients (disease duration > 5 years) were untreated at the time of biopsy, consistent with the more quiescent nature of the disease in this population. *Rhodotorula* sequences were consistently detected in both SSc and controls, though these levels never rose above the background noise. Such an observation indicates that while *Rhodotorula* may be increased in very early disease, colonization does not persist over time.

Few viral pathogens were detected in our cohort, with no reads associated with EBV, parvovirus B19, or CMV identified in lesional skin. In contrast, we did consistently identify sequences associated with *Roseolovirus*, a genus which contains both human Herpesviruses (HHV) 6 and 7, which exhibited modest increases in inflammatory SSc patients. As EBV (HHV4), CMV (HHV5), and *Roseolovirus* are all members of the Herpesvirus family, detection of active viral transcription in inflammatory lesional skin does suggest a potential link between life-long latent viral infections and disease pathology, though further studies will be necessary to prove such an association.

## Conclusions

The data presented here demonstrate a possible mechanistic link between SSc skin microbiome composition and disease pathology, with a loss of skin appendages and lipid signaling leading to decreases in lipophilic taxa, and a shift to a largely Gram-negative environment. Host-microbiome studies will be necessary to assess the extent to which the microbiome shapes SSc-associated gene expression and vice versa.

## Additional files

Additional file 1: **Table S1.** Gene expression data. Normalized RNA-seq data were classified using a support vector machine trained using a merged and curated dataset composed of samples from GSE9285, GSE32413, and GSE45485. To visual results, the probe ID list from Johnson et al. [4] was collapsed on gene ID. This gene list was compared against normalized FPKM values for all 36 RNA-seq samples, resulting in a total of 1010 overlapping genes. Intrinsic subset assignments for individual genes are not possible based on the nature of gene expression in SSc patients. Each patient’s intrinsic subset assignment was determined based on the collective co-expression of all 1010 genes in this dataset, with both high and low expression of individual genes important for determining subset distinctions. Furthermore, both high and low expression of individual genes often extends across multiple intrinsic subsets. This inherently prevents providing subset-specific calls for individual genes. (XLSX 418 kb)

Additional file 2: **Table S2.** Full clinical data for all patients included in this study. (XLSX 14 kb)

Additional file 3: **Table S3.** Antimicrobial gene expression in lesional and control skin. (XLSX 36 kb)

Additional file 4: **Table S4.** Differences in genus-level abundance by clinical covariate. Statistical analyses were performed comparing genus-level abundance between groups, presented as *p* values. Data were compared using the Mann-Whitney U test, corrected for multiple hypothesis testing using the method of Benjamini & Hochberg. Statistically significant differences (*p* < 0.05) are highlighted in yellow; differences significant to *p* < 0.10 are shown in pink. (XLSX 24 kb)

Additional file 5: **Figure S1.** Principal component analysis of lesional forearm samples based on mRSS. Principal component analysis of core microbiome profiles based on mRSS. Data were limited to SSc lesional forearm samples only. Patients were divided into quartiles based on mRSS score at the time of biopsy (low, < 5; medium, 6–15; high, 16–30; very high, > 30). (PPTX 77 kb)

Additional file 6: **Figure S2.** Principal component analysis of paired lesional forearm samples. Core microbiome profiles from seven paired forearm and back samples were analyzed by principal component analysis to assess the relationship between anatomical sites. Samples were color coded by A) anatomical site, and B) patient. (PPTX 91 kb)

Additional file 7: **Table S5.** Single-sample gene set enrichment analysis in SSc patients. Single-sample gene set enrichment analysis (ssGSEA) [26] was run as a module in GenePattern, using relevant KEGG pathways as the query gene sets. Raw pathway enrichment scores are shown for each sample in our dataset. (XLSX 102 kb)

Additional file 8: **Figure S3.** Comparing gene expression with taxonomic abundance. Single-sample gene set enrichment analysis (ssGSEA) provides a quantitative measurement, expressed as a single value, describing the extent to which a given gene set is coordinately up- or downregulated in a sample. A. To reduce the dimensionality of the data, the activation of a given KEGG pathway was assessed using ssGSEA, reducing a large set of functionally related genes to a single value for each patient. This process was repeated for all available KEGG pathways, generating a table of pathway activation scores for each patient sample (Additional file 2: Table S2). B. Pearson’s correlations were then used to compare each set of pathway activation scores against the relative abundance of each genus in the SSc skin core microbiome. C. This process is repeated for each combination of KEGG pathway and genus, producing a correlation matrix. D. Data are then clustered hierarchically and visualized to identify patterns of gene expression, and its relationship to microbial abundance. (PPTX 113 kb)

Additional file 9: **Figure S4.** Differences in kingdom- and phylum-level abundance between groups. Kingdom- and phylum-level abundance is shown for all major gene expression clusters (Fig. 4a, b). Data are presented as log2 median-centered values for all forearm biopsies from SSc (blue) and controls (gray). (TIF 7050 kb)

## Abbreviations

AD: Atopic dermatitis
CMV: Cytomegalovirus
dSSc: Diffuse systemic sclerosis
EBV: Epstein-Barr virus
FPKM: Fragments per million mapped reads
IMSA: Integrated metagenomic sequencing analysis
lSSc: Limited systemic sclerosis
NCBI: National Center for Biotechnology Information
QIIME: Quantitative Insights Into Molecular Ecology
SPSS: Statistical Package for Social Sciences
SSc: Systemic sclerosis
ssGSEA: Single-sample gene set enrichment analysis
TGFβ: Transforming growth factor beta
TLR: Toll-like receptor
