# Supplementary figures and images for: Microbiome dysbiosis is associated with disease duration and increased inflammatory gene expression in systemic sclerosis skin
Source: Arthritis Res Ther. 2019 Feb 6;21:49. doi: 10.1186/s13075-019-1816-z (PMC6366065; doi:10.1186/s13075-019-1816-z)

## Slide 1
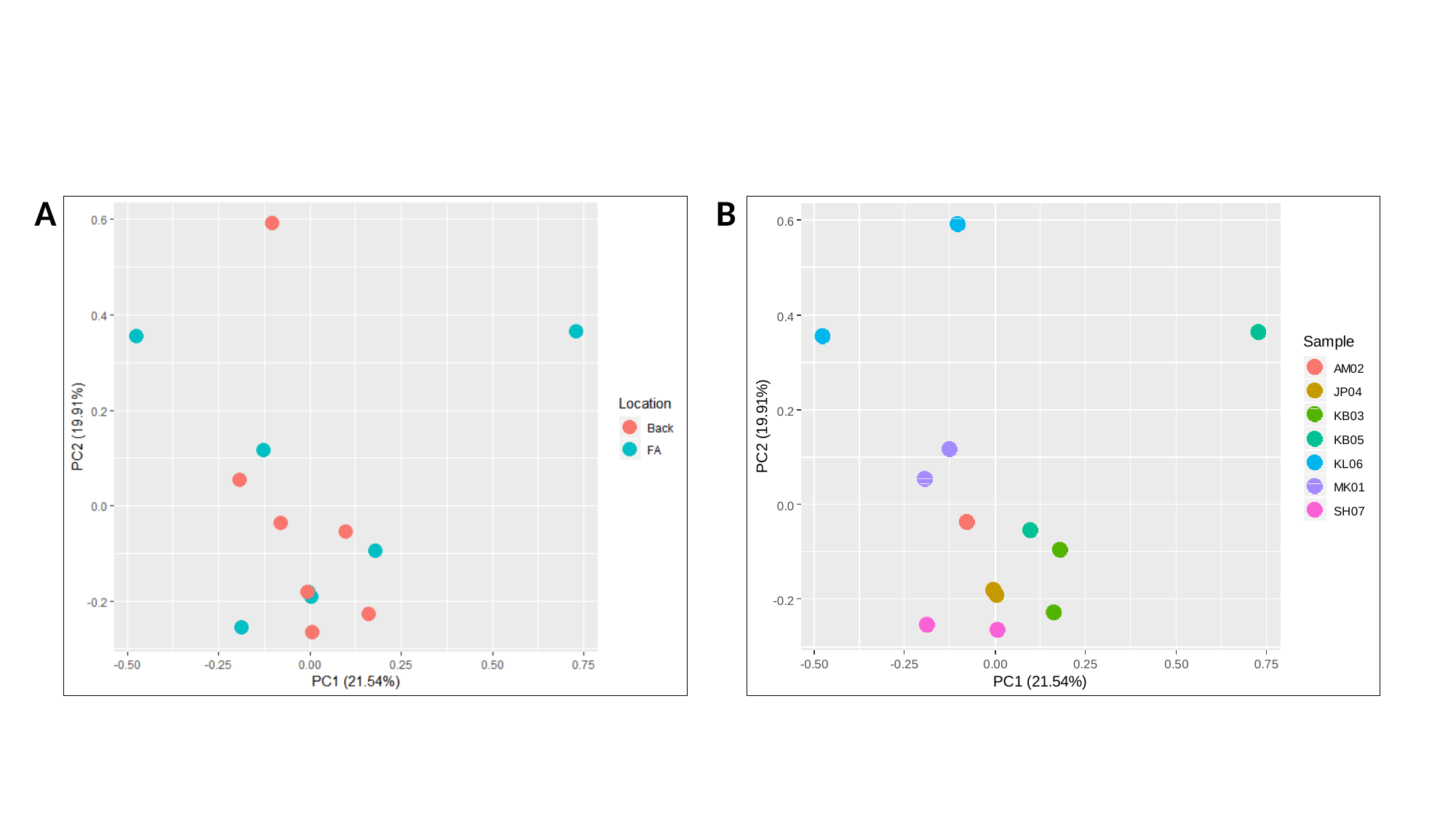

A
B

Supplement: Supplementary file 5 — Figure S1. Principal component analysis of lesional forearm samples based on mRSS. Principal component analysis of core microbiome profiles based on mRSS. Data were limited to SSc lesional forearm samples only. Patients were divided into quartiles based on mRSS score at the time of biopsy (low, < 5; medium, 6–15; high, 16–30; very high, > 30). (PPTX 77 kb) [file 13075_2019_1816_MOESM5_ESM.pptx]

## Slide 1
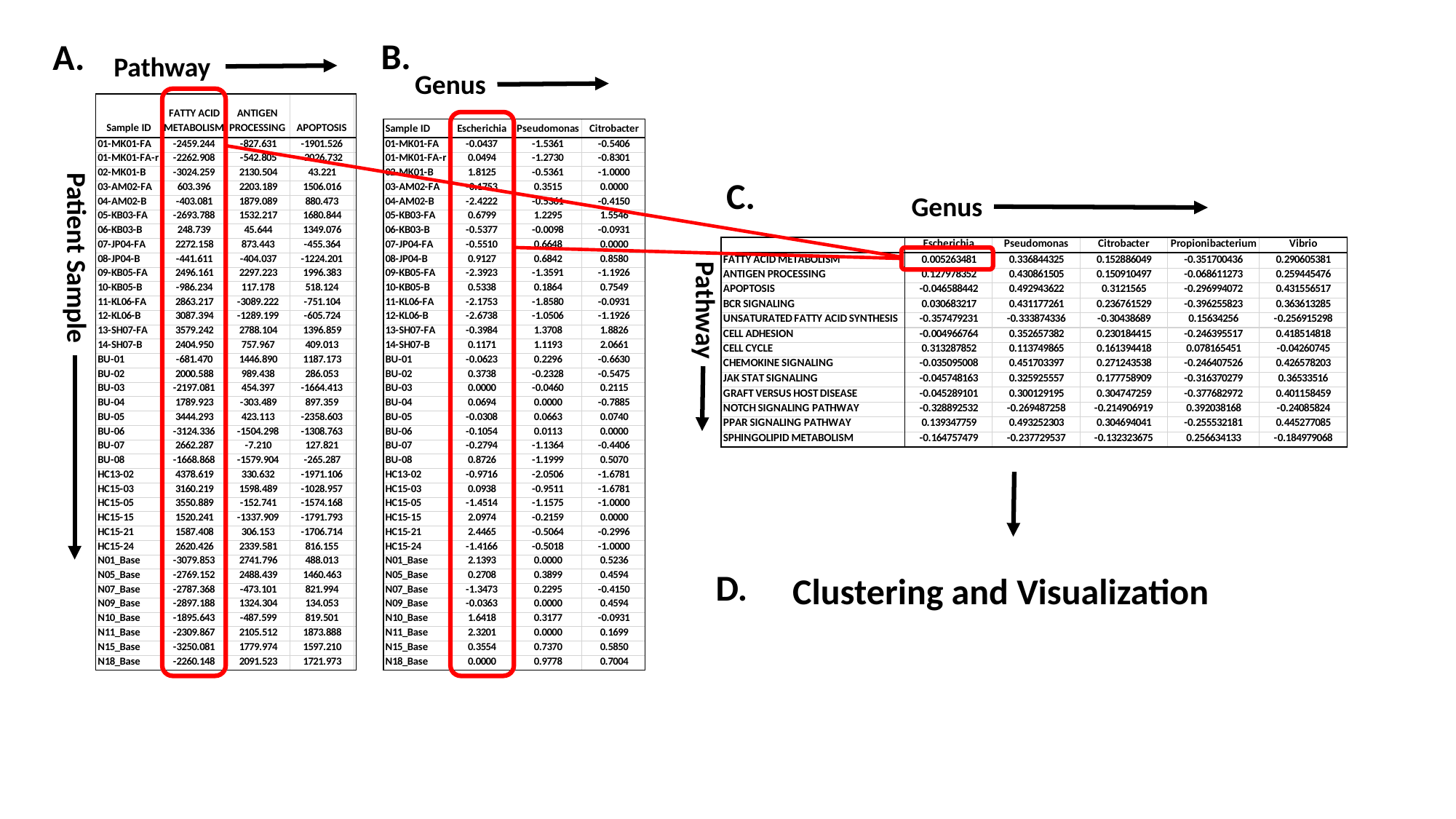

B.
A.
Pathway
Genus
C.
Genus
Patient Sample
Pathway
D.
Clustering and Visualization

Supplement: Supplementary file 6 — Figure S2. Principal component analysis of paired lesional forearm samples. Core microbiome profiles from seven paired forearm and back samples were analyzed by principal component analysis to assess the relationship between anatomical sites. Samples were color coded by A) anatomical site, and B) patient. (PPTX 91 kb) [file 13075_2019_1816_MOESM6_ESM.pptx]
