# Supplementary material for: Microbiome dysbiosis is associated with disease duration and increased inflammatory gene expression in systemic sclerosis skin
Source: Arthritis Res Ther. 2019 Feb 6;21:49. doi: 10.1186/s13075-019-1816-z (PMC6366065; doi:10.1186/s13075-019-1816-z)
Supplement: Supplementary file 8 — Figure S3. Comparing gene expression with taxonomic abundance. Single-sample gene set enrichment analysis (ssGSEA) provides a quantitative measurement, expressed as a single value, describing the extent to which a given gene set is coordinately up- or downregulated in a sample. A. To reduce the dimensionality of the data, the activation of a given KEGG pathway was assessed using ssGSEA, reducing a large set of functionally related genes to a single value for each patient. This process was repeated for all available KEGG pathways, generating a table of pathway activation scores for each patient sample (Additional file 2: Table S2). B. Pearson’s correlations were then used to compare each set of pathway activation scores against the relative abundance of each genus in the SSc skin core microbiome. C. This process is repeated for each combination of KEGG pathway and genus, producing a correlation matrix. D. Data are then clustered hierarchically and visualized to identify patterns of gene expression, and its relationship to microbial abundance. (PPTX 113 kb) [file 13075_2019_1816_MOESM8_ESM.pptx]

## Slide 1
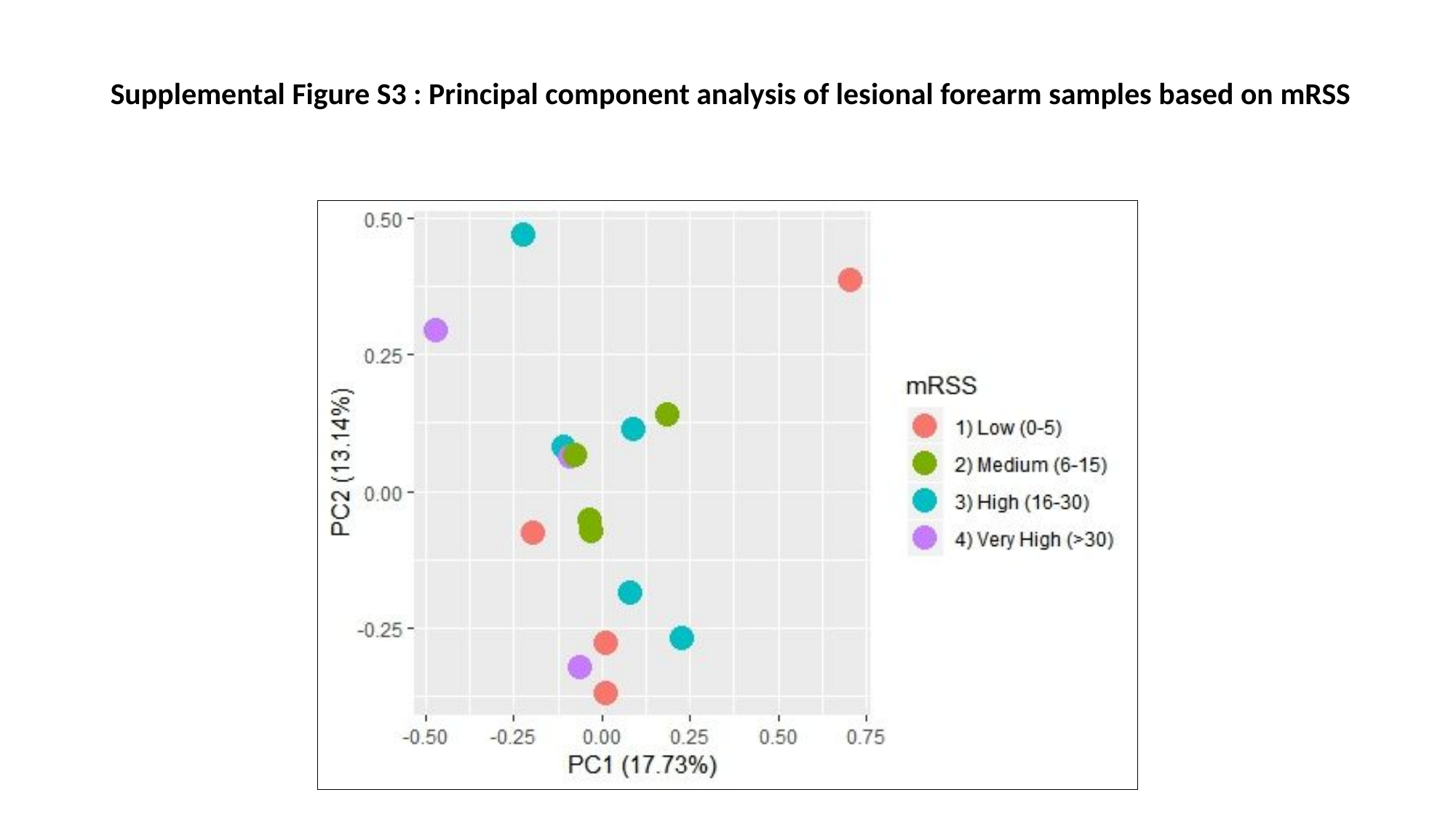

Supplemental Figure S3 : Principal component analysis of lesional forearm samples based on mRSS
